# Global Leadership Initiative on Malnutrition Criteria Predict Pulmonary Complications and 90-Day Mortality after Major Abdominal Surgery in Cancer Patients

**DOI:** 10.3390/nu12123726

**Published:** 2020-12-03

**Authors:** Sotirios Kakavas, Dimitrios Karayiannis, Zoi Bouloubasi, Kalliopi Anna Poulia, Steven Kompogiorgas, Dimitrios Konstantinou, Vasileios Vougas

**Affiliations:** 1 1st Pulmonary Department, Evangelismos General Hospital of Athens, Ypsilantou 45-47, 10676 Athens, Greece; sotikaka@yahoo.com (S.K.); stevenkompogiorgas@googlemail.com (S.K.); 2 Department of Clinical Nutrition, Evangelismos General Hospital of Athens, Ypsilantou 45-47, 10676 Athens, Greece; zoippp@yahoo.com; 3 Department of Nutrition and Dietetics, Laiko General Hospital, 11527 Athens, Greece; lpoulia@gmail.com; 4 Transplant Unit, 1st Department of Surgery, Evangelismos General Hospital of Athens, 10676 Athens, Greece; dimikonan@yahoo.com (D.K.); drvougas58@yahoo.gr (V.V.)

**Keywords:** malnutrition, abdominal surgery, cancer, postoperative complications

## Abstract

Although several studies have reported an association between malnutrition and the risk of severe complications after abdominal surgery, there have been no studies evaluating the use of Global Leadership Initiative on Malnutrition (GLIM) criteria for predicting postoperative pulmonary complications (PPCs) following major abdominal surgery in cancer patients. This study aimed to investigate the association among the diagnosis of malnutrition by GLIM criteria, PPCs risk and 90-day all-cause mortality rate following major abdominal surgery in cancer patients. We prospectively analyzed 218 patients (45% male, mean age 70.6 ± 11.2 years) with gastrointestinal cancer who underwent major abdominal surgery at our hospital between October 2018 and December 2019. Patients were assessed preoperatively using GLIM criteria of malnutrition, and 90-day all-cause mortality and PPCs were recorded. In total, 70 patients (32.1%) were identified as malnourished according to GLIM criteria, of whom 41.1% fulfilled the criteria for moderate and 12.6% for severe malnutrition. PPCs were detected in 48 of 218 patients (22%) who underwent major abdominal surgery. Univariate logistic regression analysis revealed that the diagnosis of malnutrition was significantly associated with the risk of PPCs. Furthermore, in multivariate model analysis adjusted for other clinical confounding factors, malnutrition remained an independent factor associated with the risk of PPCs (RR = 1.82; CI = 1.21–2.73) and 90-day all-cause mortality (RR = 1.97; CI = 1.28–2.63, for severely malnourished patients). In conclusion, preoperative presence of malnutrition, diagnosed by the use of GLIM criteria, is associated with the risk of PPCs and 90-day mortality rate in cancer patients undergoing major abdominal surgery.

## 1. Introduction

The detrimental effects of malnutrition on patients’ outcomes have been reported in various diseases and clinical settings, for both outpatients and hospitalized patients [1,2,3,4,5]. This is especially true for cancer patients who are considered as a patient group highly affected by nutrition deficiencies and high risk of malnutrition [6,7,8]. Bothtumor and anticancer treatment have a negative impact on nutritional status of these patients and pose a particularly high risk for malnutrition [6]. Indeed, cancer cachexia is present in 50–80% of cancer patients and leads to the death of at least 20% of them [7,9]. Cancer-related malnutrition (CRM) is driven by a multifactorial and complex set of mechanisms, leading to decreased physical function, impaired tolerance to treatment, increased toxicity, decreased quality of life and eventually decreased survival. However, the risk of CRM is sometimes underdiagnosed and undertreated, especially when screening is based solely on body mass index (BMI) and weight history, as 40–60% of the patients are overweight or obese, even in the setting of a metastatic disease, something that can mask the risk of malnutrition [9]. Thus, guidelines suggest screening all cancer patients for nutritional risk early enough during their management by using more composite tools [6].

Disease-related malnutrition (DRM) is a condition characterized by impaired intake and/or assimilation of nutrients, which leads to changes in body composition and reduced functional capacity. Recently, the Global Leadership Initiative on Malnutrition (GLIM) criteria for the diagnosis of malnutrition have been proposed by the European Society for Clinical Nutrition and Metabolism (ESPEN), in an attempt to build a global consensus around core diagnostic criteria for malnutrition in adults in different clinical settings [10,11]. Inhospitalized patients, the presence of malnutrition hinders wound healing as well as the development of an effective response to infection [5]. Therefore, it is not surprising that the risk of infectious and other complications during hospitalization and the risk of adverse outcomes after discharge are increased in malnourished patients [6,12]. Similarly, in surgical patients, malnutrition has been shown to increase postoperative complication rate, mortality, length of hospital stay (LOS) and subsequently costs [13,14]. In relation to postoperative complications, previous studies correlate malnutrition to overall postoperative complications rate—on the principle of grading complications—without focusing on any specific type of complication [15,16]. Postoperative pulmonary complications (PPCs), encompassing complications affecting the respiratory system after anesthesia and surgery, represent one of the main causes of postoperative morbidity and mortality after abdominal surgery [17,18] and their incidence ranges from 1% to 23% [19,20,21]. Previous studies have evaluated multiple ways of assessing nutritional status in surgical patients and their association to postoperative complications and mortality. Although recent data indicate that preoperative presence of malnutrition according to GLIM criteria [22,23,24,25] is associated with an increased risk of severe surgical complications [21], thusfar, no study has tested the predictive power of malnutrition on PPCs and all-cause mortality. Given the high prevalence of malnutrition in cancer patients and the magnitude of PPCs after abdominal surgery, a possible association could be hypothesized. If this notion were to be verified, it would further add to the significance of prompt screening for malnutrition risk in this special group of patients as an additional strategy to minimize PPCs and associated mortality.

This study aimed to explore the independent prognostic ability of GLIM criteria to predict primarily PPCs and secondarily 90-day all-cause mortality rate in cancer patients who underwent elective open abdominal surgery after adjusting for other clinically relevant variables.

## 2. Methods

### 2.1. Study Participants and Design

The prospective observational study included eligible patients with cancer who were posted for elective abdominal surgery in the time from October 2018 to December 2019. Inclusion criteria comprised patients aged greater than 18 years, with a previous diagnosis of a solid neoplasm and a scheduled elective open abdominal surgery. The exclusion criteria were emergency surgeries and patient’s denial to provide a written informed consent or inability to collect complete nutritional information. Collectively, 218 recruited patients with gastrointestinal cancer who were submitted to elective abdominal surgery were prospectively analyzed. Elective surgery was defined as start of anesthesia between 7 am and 4 pm. All patients received standard clinical care both preoperatively and postoperatively. Study was approved by the Institutional Ethics Committee of Evangelismos General Hospital of Athens (a tertiary hospital, ClinicalTrials.gov identifier: NCT03719508), and data were consecutively collected between October 2018 and December 2019. Within the first 24 h of hospitalization, all study participants provided informed, written consent.

### 2.2. Measures and Data Collection

At baseline, basic demographic data were obtained with a structured clinical history and physical examination. Preoperative weight and height were measured with a calibrated scale and stadiometer (SECA764 Scale), and Body Mass Index (kg/m^2^) was calculated. Recorded data included age, gender, previous co-morbidities, oncological diagnosis, weight loss in the past 3–6 months (self-reported) and dietary intake during the last preadmission week. All measurements were carried out by the primary dietitian of the Unit’s Nutrition Support Team. Complete blood counts and basic biochemical values were measured by standard laboratory methods from blood samples obtained by peripheral vein shortly after the admission of patients. Malnutrition presence was assessed using the following GLIM phenotypic and etiologic criteria [10]:

(1) Unintentional weight loss (>5% in 6 months);
(2) Patient had low BMI (<20 kg/m^2^ if <70 years and<22 kg/m^2^ if ≥70years); or
(3) Reductionof muscle mass based on calf circumference wasused as phenotypic criteria. For this test, knee was flexed to 90° with the feet and ankles relaxed, and the largest calf circumference was measured using a standard tape measure with 0.1 cm increment. Values lower than 31 cm were considered as low [26].
(4) Reduced intake (>50% of energy intake during the last pre-admission week) orinflammatory response of the disease (chronic disease-related inflammation was evaluated using C reactive protein (CRP) values >5mg/dL) was used as etiologic criteria.

To diagnose malnutrition, at least one phenotypic criterion and one etiologic criterion had to be present. Severely malnourished patients were defined those who presented weight loss >10% within the past six months; BMI < 18.5 m/kg^2^ (<70 years) or BMI < 20 m/kg^2^ (≥70 years); or calf circumference values < 31 cm. Patients with moderate malnutrition were defined those with weight loss 5–10% within the past sixmonths and/or BMI 18.5–20 m/kg^2^ (age < 70 years) or BMI < 22 m/kg^2^ (≥70 years).

### 2.3. Outcomes

Patients were followed up postoperatively during the time of hospitalization. The primary outcome of the present study was the occurrence of in hospital PPCs. The presence of PPCs was assessed by an experienced chest physician based on appropriate clinical, laboratory and radiological data and arterial blood gases [27]. By these means, PPCs were identified and classified as: acute respiratory failure, bronchospasm, pulmonary embolism, pneumothorax, atelectasis, pleural effusion, tracheobronchitis, pneumonia, acute respiratory distress syndrome and prolonged mechanical ventilation (>48 h). Secondary outcomes included all cause 90-day all-cause mortality and length of hospital stay (LOS). LOS was defined as the period (in days) from hospital admission to hospital discharge. If needed, postoperative evaluation for primary and secondary outcomes was carried out both in the intensive care unit (ICU) stay and on the general ward.

### 2.4. Statistical Analysis

Statistical analysis was performed using the statistical software SPSS version 21.0 (SPSS, Inc., Chicago, IL, USA). For sample size calculation, a prevalence of about 20% of postoperative pulmonary complications in patients undergoing major abdominal surgery was used [25]. The estimated required sample size to have approximately 80% power at α = 0.05 to detect a 20% difference of PPCs between those being malnourished and those not (according to GLIMM criteria) was 190 patients. Continuous variables are presented as mean (±standard deviation) and categorical variables as absolute and relative frequencies. Associations between categorical variables were tested by χ^2^ tests or Fisher’s exact tests (when one or more cell counts were ≤5). Assumption of normal distribution of continuous variables was assessed by the Kolmogorov–Smirnov test. For continuous quantitative variables, means comparison was performed by the Student’s t-test for variables normally distributed and the unpaired-samples Mann–Whitney rank sum test for non-parametric variables. The possibility of a linear association between calf circumference and various measures was assessed by Pearson’s correlation coefficient (r). LOS was found to be highly skewed, therefore the logarithmic transformation of LOS was used. Univariate logistic regression analyses were performed to examine the association between the outcome of interest (PPCs—90-day mortality rates) and each of the predictors separately in order to identify factors significantly associated with an increased risk of PPCs or death; for each variable, the relative risk (RR) and 95% confidence interval (CI) are given. We used generalized linear models with binomial distribution and logit link function to test associations between malnutrition status (yes vs. no) and PPCs or 90-day all-cause mortality rates. The results are presented in terms of relative risk (RR) and 95% CIs. Confounding was evaluated using prior knowledge regarding biological relevance as well as descriptive statistics from our study population. In all analyses, a two-sided *p* value less than 0.05 was considered statistically significant.

## 3. Results

**Figure 1:**
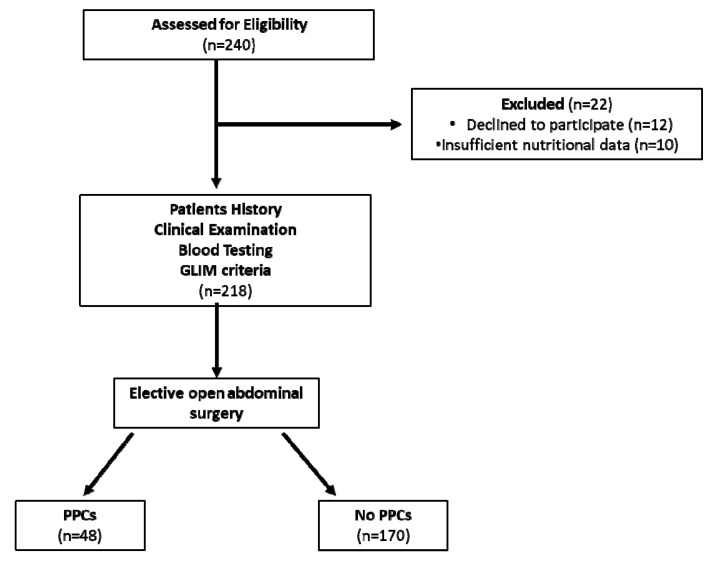
Flowchart of enrolled patients. GLIM, Global Leadership Initiative on Malnutrition; PPCs, postoperative pulmonary complications.

During the study period, 240 patients were initially assessed for eligibility. Twelve patients were excluded because they declined participation and 10 patients were excluded due to missing nutritional assessment data (no weight loss or dietary intake data). In total, 197 patients were elective admissions specifically to undergo abdominal surgery, whereas 21 patients were admitted to hospital for gastrointestinal diagnostic tests, found to have gastrointestinal cancer and then scheduled for surgery. All enrolled patients underwent elective abdominal surgery and were subsequently divided into subgroups during the postoperative follow-up, according to the occurrence of PPCs (Figure 1).

Data from 218 patients were analyzed. Patients’ baseline and postoperative demographic, clinical and laboratory data are reported in Table 1. Table 1 also provides the comparison of characteristics of patients with and without PPCs. Our final sample consisted of 90 (49%) males and 128 (55%) females, with a mean age of 70.1 ± 13.1 years. Median preoperative LOS (number of days from hospital admission until the day of surgery) was three days (2–5), while 7.2% of patients underwent preoperative nutritional therapy, after a detailed nutritional assessment (provision of enteral or parenteral nutrition). Seventy-four patients (34%) reported at least one underlying medical condition apart from cancer. According to GLIM criteria, malnutrition was diagnosed in 72 patients (33%), of whom 17.4% were moderately malnourished and 13.6% severely malnourished. On the other hand, 118 patients reported reduced dietary intake preoperatively. The most frequent PPCs were pneumonia (11%), pleural effusion (10%), atelectasis (11%) and prolonged mechanical ventilation (16%). Other PPCs were observed in 5.5% of the patients. As shown in Table 1, patients with PPCs were characterized by more frequent co-morbidities, increased prevalence of malnutrition, prolonged LOS and increased in hospital mortality. In addition, patients with PPCs had higher prevalence of malnutrition at time of surgery and were more likely to die within 90 days, as compared to those who were not (all *p* < 0.05). Of all patients admitted for surgery, 110 (50.4%) were supported with early Enteral Nutrition (EN) (<48 h) and 76 (34.8%) received late EN (>48 h). A small subset of 32 patients received parenteral nutrition. Overall, 33.2% of patients were admitted to intensive care unit (ICU) for postoperative care for average length of 4.2 days (±3.2), after which they were transferred back to the surgical ward.

Regarding the diagnosis of malnutrition, median LOS was statistically significantly longer in malnourished patients (29.2 vs. 17.2), while 90-day all-cause mortality rates were also higher (30.5% vs. 11.4%). Calf circumference was positively and significantly correlated to serum albumin (sAlb) levels (Spearman rho = 0.254, *p* = 0.005), while age (rho = −0.216, *p* = 0.020) and CRP levels (rho = −0.298, *p* = 0.031) were negatively correlated.

Table 2 summarizes the results of univariate logistic regression analysis of the relationship between the occurrence of PPCs and multiple predictors. The risk of PPCs was significantly associated with the preoperative plasma levels of urea and creatinine, the presence of pulmonary co-morbidities and preoperative existence of malnutrition according to GLIM criteria.

However, neither the absence nor the presence of malnutrition was predictive of in hospital all-cause mortality (RR = 1.32; CI = 0.61–2.04). We then examined the relation of malnutrition with PPCs and 90-day all-cause mortality. Variables with *p* ≤ 0.05 during univariate analysis were included in a multivariate model to identify factors with independent predictive value for PPCs and death. The multivariable-adjusted RR (95% CI) for PPCs comparing moderate/severely malnourished to non-malnourished patients (Table 3) revealed that preoperative presence of malnutrition was independently associated to increased risk of PPCs and 90-day all-cause mortality (RR = 1.82; CI = 1.21–2.73; and 1.97; CI = 1.28–2.63 in moderately and severely malnourished patients, respectively) even after controlling for multiple possible confounders.

## 4. Discussion

The present prospective study evaluated the prognostic ability of GLIM criteria for the prediction of pulmonary complications in cancer patients who underwent major abdominal surgery. The presence of malnutrition, as diagnosed by GLIM criteria, was an independent predictor of PPCs and 90-day mortality rate. This study is the first to our knowledge evaluating the predictive ability of GLIM criteria for the occurrence of PPCs in a population submitted to abdominal surgery for cancer. Previous studies have shown that malnutrition is associated with increased postoperative complication rate, mortality rate and LOS [13,14,24] and that PPCs enhance postoperative morbidity and mortality after abdominal surgery [15,17,18]. The latter is consistent with current study findings, since, according to our results, patients with PPCs were characterized by prolonged LOS and increased in hospital and 90-day all-cause mortality. Furthermore, malnourished patients presented a prolonged LOS and increased risk of in hospital all-cause mortality.

A significant proportion (33%) of patients in the present study were found to meet GLIM criteria for malnutrition. This is not surprising since the high prevalence of malnutrition in cancer patients is well established. Previous studies have reported a prevalence of malnutrition in patients with cancer as high as 80% [7,28]. Cancer cachexiais a multifactorial and complex syndrome in which pro-inflammatory cytokines and specific tumor-derived factors enhance an energy-intensive acute phase protein response [7]. This process is reflected in our results for lower sAlb and raised CRP levels in malnourished patients. In addition, sAlb was associated with PPCs risk in univariate analysis. In previous studies, preoperative sAlb levels were considered to be a risk factor for postoperative morbidity, including PPCs, and mortality after oncologic elective abdominal surgery [29,30]. Patients with PPCs also showed lower hemoglobin levels. In cancer patients, anemia is common and has been associated with worse postoperative outcomes [31]. Preoperative anemia has been previously recognized as a predictor of PPCs [32].

Moreover, our analysis revealed malnutrition as a preoperative marker that predicts the risk of PPCs. Earlier studies have shown that malnutrition, assessed by validated tools, is associated with prolonged LOS and increased morbidity and mortality in patients undergoing major abdominal surgery [24,25,33]. Most importantly, in our study, the predictive ability of impaired nutritional status remained significant even after controlling for possible confounders. The exact mechanism by which malnutrition is associated with PPCs remains unknown. The malnutrition induced decrease in muscle mass may be associated to the occurrence of PPCs, through a decline in respiratory muscle strength [34], lung atelectasis and pneumonia incidence [35]. Regarding mortality, recent findings support the hypothesis that low muscle mass is also associated with the possibility of death after major surgery [36]. In the present study, calf circumference was strongly and positively correlated with sAlb levels, which has been in turn associated with a higher likelihood of death [37]. Considering also that malnutrition is a state of fat mass reduction, which is not only a storage depot, but also a nutritional reserve that influences the inflammatory and immune response, we may hypothesize that malnourished patients present a decreased ability to handle the stress and nutritional cost of major surgery [38].

It should be noted that, in our results, neither BMI impairment nor weight loss six months before surgery was associated with the occurrence of PPCs. These findings may contradict part of the available literature concerning surgical patients [39,40], however they seem to agree with the recommendations of the ESPEN oncology expert group according to which all cancer patients should be screened early for nutritional risk regardless of BMI and weight history [6]. Furthermore, early screening and appropriate nutritional therapy should be offered to surgical patients with increased nutritional risk to reduce postoperative morbidity and mortality [41]. Thus, by extrapolating these suggestions, proper nutritional assessment and intervention before abdominal surgery in cancer patients may also reduce the incidence of PPCs. Malnutrition diagnosis using GLIM criteria seems to fulfill adequately its preoperative role for the identification of patients who would benefit from the aforementioned strategy. Moreover, the current study indicates that malnutrition grading in the GLIM criteria (moderate and severe forms) is appropriate in the clinical setting, since the criteria for severe malnutrition tends to be more strongly associated with severe postoperative complications and 90-day all-cause mortality, as compared to the criteria for moderate malnutrition.

Our study design has several limitations. First, regarding 90-day all-cause mortality as outcome measure, the study sample size was small, resulting in a limited number of overall deaths. This makes it difficult to extend the findings to the general patient population and should be taken into account when evaluating the prognostic value of GLIM criteria on postoperative mortality, although data from a recent study confirm a supporting opinion that malnutrition according to GLIM criteria is associated to a higher probability of death among cancer inpatients [40,42]. Secondly, due to the study’s prospective design, it should be mentioned that we are able to only disclose associations and not infer causality. Third, we were unable to assess muscle mass using a validated indicator such as bioelectrical impedance analysis (BIA), computed tomography (CT) or magnetic resonance imaging (MRI), so a standard anthropometric measure—calf circumference—was used as a surrogate marker of muscle mass. The consensus report about GLIM criteria recommends that, in situations where muscle mass cannot be readily assessed, physical examination or standard anthropometric measures such as calf circumferences may be used [10]. Finally, apart from the type of surgery, we were unable to collect other intraoperative data including duration of operation or other therapeutic interventions. Therefore, it is probable that differences in the intraoperative characteristics could have influenced postoperative outcomes.

## 5. Conclusions

The present study explored the connection between preoperative nutritional status and postoperative PPCs and mortality rate in cancer patients undergoing major abdominal surgery. Malnutrition according to GLIM criteria was present in almost one-third of patients preoperatively. These patients were characterized by a prolonged LOS. Multivariate models adjusted for major clinical variables revealed that malnutrition was significantly associated to postoperative PPCs risk and remained an independent predictor of 90-day all-cause mortality risk. These results further underline the importance of preoperative nutritional screening and assessment and the prompt provision of nutritional support in surgical candidates and especially in cancer patients.

## Figures and Tables

**Table 1:** Patients demographics, baseline and postoperative characteristicsaccording to postoperative pulmonary complications occurrence (PPC).

Characteristic	Total (*n* = 218)	PPCs (*n* = 48)	No PPCs (*n* = 170)	*p*-Value
Age (years)	70.1 ± 13.1	73.9 ± 9	69 ± 13.9	0.23
Sex, male, *n* (%)	90 (49)	21 (43.7)	69 (40.5)	0.30
Albumin (g/dL)	3.87 ± 0.6	3.34 ± 0.58	3.93 ± 0.59	<0.05
CRP (mg/dL)	2.46 ± 3.7	3.4 ± 4.5	2.2 ± 3.5	0.13
Hb (g/dL)	11.5 ± 1.9	10.9 ± 1.8	11.7 ± 1.9	0.07
Urea (mg/dL)	42.9 ± 18	55.2 ± 23.6	39.5 ± 14.6	<0.001
Creatinine (mg/dL)	0.95 ± 0.6	1.25 ± 1.2	0.86 ± 2.4	<0.05
Comorbidities *, *n* (%)	68 (31.1)	30 (62.5)	38 (22.3)	<0.001
Respiratory comorbidities	22 (10)	14 (29)	8 (4.7)	<0.001
Cardiovascular comorbidities	34 (15.6)	18 (37.5)	16 (9.4)	<0.01
Metabolic comorbidities	32 (14.7)	24 (50)	8 (4.8)	<0.001
BMI <20 if <70 years or <22 if >70 years	36 (16.5)	8 (16.6)	28 (16.5)	0.98
Weight loss in past 3–6 months, *n* (%)	122 (56)	36 (75)	86 (50.6)	<0.01
<5%	96 (44)	12 (25)	84 (49.4)	
5–10%	74 (34)	24 (50)	50 (29.4)	
>10%	48 (22)	12 (25)	36 (21.2)	
Reduced dietary intake in past week	118 (54)	28 (58.3)	90 (53)	0.64
Diagnosis of Malnutrition, *n* (%)	72 (33.0)	30 (62.5)	42 (24.7)	<0.001
Moderate Malnutrition, *n* (%)	38 (17.4)	16 (33.3)	22 (12.9)	<0.01
Severe Malnutrition, *n* (%)	30 (13.7)	14 (29.1)	16 (9.5)	<0.01
Underlying condition, n (%)				0.76
Gastric cancer	44 (20)	14 (29.2)	30 (17.6)	
Pancreatic cancer	22 (10)	8 (16.6)	14 (8.2)	
Hepatic cancer	12 (5.5)	4 (8.3)	8 (4.7)	
Colorectal cancer	128 (59)	26 (54.2)	102 (60)	
Type of surgical procedure				0.29
Conventional	168 (77.0)	38 (80.5)	130 (74.1)	
Laparoscopic	50 (23.0)	9 (19.5)	41 (24.1)	
Colorectal resection	78 (35.7)	18 (38.5)	60 (35.2)	
Gastric Resection	30 (13.8)	4 (9.6)	26 (17.2)	
Small bowel resection	29 (13.3)	7 (14.4)	22 (12.8)	
Hepatobiliary resection	26 (11.9)	7 (14.4)	19 (8.7)	
Other **	55 (25.3)	13 (27.1)	42 (24.7)	
Length of hospital stay (days)	20.2 (10.2–30.7)	26.7 (11.3–31.5)	18.2 (7.3–32.3)	0.02
In hospital death, *n* (%)	12 (5.5)	8 (16.6)	4 (2.3)	<0.01
90-day all-cause mortality, *n* (%)	44 (20.1)	24 (50)	20 (11.7)	<0.01

Values represent mean values (± SDs), median (25th–75th percentile) or number of subjects (*n*, %). Differences between groups (PPCs vs. no PPCs) were tested using chi square (categorical) test or Student’s t test (continuous variables). * Respiratory comorbidities: chronic obstructive pulmonary disease and airway infections; Cardiovascular comorbidities: heart failure, coronary artery disease, peripheral vascular disease; Metabolic comorbidities: diabetes, obesity, dyslipidemia, metabolic syndrome. ** Other: esophageal resection, cholecystectomy, pancreatic resection, CRP, c reactive protein.

**Table 2:** Univariate logistic regression analysis of predictive factors associated with postoperative pulmonary complications (PPCs) and 90-day all-cause mortality after major abdominal surgery in patients with cancer.

	PPCs			90-Day All-Cause Mortality		
Variables	Relative Risk	95% CI	*p*	Relative Risk	95% CI	*p*
Sex (Male vs. Female)	1.61	0.64–4.00	0.30	1.42	1.08–1.76	0.02
Age (years)	1.03	0.99–1.08	0.11	1.75	1.09–2.42	0.03
Albumin (g/dL)	0.44	0.20–0.94	0.03	4.24	2.20–6.19	0.01
C-reactive protein (mg/dL)	1.07	0.96–1.20	0.18	1.07	0.96–1.20	0.18
Hb (g/dL)	0.81	0.63–1.03	0.08	0.82	0.58–1.06	0.13
Urea (mg/dL)	1.04	1.02–1.07	<0.01	1.52	0.76–2.27	0.32
Creatinine (mg/dL)	5.80	1.25–26.96	0.02	3.90	0.65–7.45	0.27
Respiratory comorbidities (Yes vs. No)	4.77	1.83–12.44	<0.01	4.52	1.25–9.85	<0.01
Malnutrition (Yes vs. No)	2.34	1.66–3.04	0.01	3.24	1.52–5.14	<0.001
BMI < 20 kg/m^2^	1.01	0.30–3.42	0.98	1.23	0.60–1.93	0.11
Preoperative weight loss (%)	1.57	0.88–2.79	0.12	1.85	0.44–3.29	0.42
Reduced dietary intake in past week (Yes vs. No)	1.24	0.49–3.11	0.64	1.65	0.62–2.68	0.32

CI, Confidence Interval; Hb, Hemoglobin; BMI, Body Mass Index.

**Table 3:** Associations between malnutrition (yes vs. no) and postoperative pulmonary complications or death within 90 days after major abdominal surgery in patients with cancer (*n* = 218).

Variables	Relative Risk	95% CI	*p* Value
PPCs *			
Moderate Malnutrition			
Model I	1.72	1.26–2.18	<0.01
Model II	1.61	1.13–2.09	<0.01
Severe Malnutrition			
Model I	1.95	1.32–2.56	<0.01
Model II	1.82	1.21–2.73	<0.01
Mortality **			
Moderate Malnutrition			
Model I	1.92	1.25–2.69	<0.01
Model II	1.78	1.15–2.41	<0.001
Severe Malnutrition			
Model I	2.03	1.39–2.72	<0.01
Model II	1.97	1.28–2.63	<0.001

All analyses were conducted using generalized linear models with binomial distribution and logit link function. Data represent relative risk (95% CI). Model 1 was adjusted for age, sex and serum albumin levels. Model 2 was adjusted as for Model 1 + urea levels and preoperative respiratory comorbidities. * PPCs, postoperative pulmonary complications in 90 days; ** Mortality, 90-day mortality.

## Abbreviations

GLIM	Global Leadership Initiative on Malnutrition
BMI	Body Mass Index
PPCs	Postoperative pulmonary complications
ESPEN	European Society for Clinical Nutrition and Metabolism
LOS	Length of Hospital Stay
CRM	Cancer related malnutrition
